# Fire-Retardant Property of Hexasubstituted Cyclotriphosphazene Derivatives with Schiff Base Linking Unit Applied as an Additives in Polyurethane Coating for Wood Fabrication

**DOI:** 10.3390/polym14183768

**Published:** 2022-09-08

**Authors:** Nurul Atiqah Mohd Taip, Zuhair Jamain, Ismawati Palle

**Affiliations:** 1 Organic Synthesis and Advanced Materials (OSAM) Research Group, Faculty of Science and Natural Resources, Universiti Malaysia Sabah (UMS), Kota Kinabalu 88400, Sabah, Malaysia; 2 Faculty of Tropical Forestry, Universiti Malaysia Sabah (UMS), Kota Kinabalu 88400, Sabah, Malaysia

**Keywords:** cyclotriphosphazene, Schiff base, wood coating, fire-retardant

## Abstract

A series of new hexasubstituted cyclotriphosphaze derivatives containing Schiff base linkages were successfully synthesized and characterized. The series contains different terminal substituents of pentyl and tetradecyl. Fourier transform infrared spectroscopy (FTIR), nuclear magnetic resonance spectroscopy (NMR), and carbon, hydrogen, and nitrogen (CHN) elemental analysis were used to characterize the intermediates and final compounds, while the thermal stability of the final compounds is evaluated with a thermogravimetric analysis (TGA) test. The final compounds are physically added to the polyurethane coating formulation and then applied to the wood panel using a brush and the compound’s fire-retardant properties are evaluated using the limiting oxygen index (LOI) test. In this research, compound **3b** showed good thermal stability compared to compound **3a**. In terms of LOI results, polyurethane with an LOI value of 21.90% was employed as a matrix for wood coating and the value increased to 24.90% when this polyurethane is incorporated with 1 wt.% of the compound **3b**. The increase in the LOI value indicates that the wood coating containing hexasubstituted cyclotriphosphazene compounds exhibits excellent fire-retardant properties as additives.

## 1. Introduction

Wood has been widely used for all types of exterior and interior decoration. It is also our most plentiful renewable and environmentally benign supply due to its particular aesthetic qualities, low cost, great mechanical properties, lightweight and effectiveness [1,2,3]. However, given that its frame is comprised of a combination of carbonaceous cellulose and a three-dimensional porous structure, the inherent flammability of wood poses significant barriers to its employment in these fields [4]. According to Kong et al., 2017 [5], the protective coating of ZnO nanostructures with fire retardant was applied to the surface of the wood using a hydrothermal method. However, nanorods had covered the texture and color of the wood surface. Mahltig et al., 2008 [6], then reported numerous types of modified silica sols that had been incorporated into the wood in order to enhance its fire retardant using the sol-gel technique. However, scaling up is challenging due to the complexity of the process. However, scaling up is challenging due to the complexity of the process.

Preparation of functional coating is a very practical method for protecting wood and its products, but because the amount of fire retardants required for effectiveness is typically too high, there is a significant issue that required to be addressed regarding the compatibility of coating resins with fire retardants [7]. The application of fire-retardant coatings is intended for a variety of flammable and non-flammable surfaces. Fire retardant coatings can be recognized by their capability to release non-combustible gases when subjected to heat or flame. These gases interfere with how quickly a fire spreads and also reduce the intensity of the fire [8,9]. Therefore, improvement in the overall fire retardancy of the wood by applying a fire-retardant coating on the external surface is a good approach. Due to their production of toxic gases during combustion, the usage of halogenated fire retardants has been severely constrained. In contrast, fire retardants containing phosphorus exhibit amicable environmental protection and superior effects, both of which have gained significant attention [10,11].

Recently, most studies have been attracted to an inorganic phosphorus-nitrogen molecule known as hexachlorocyclotriphosphazene, or HCCP, which is comprised of nitrogen and phosphorus atoms that alternate with each other. This HCCP compound is connected through alternating single and double bonds [12,13]. Small structural alterations are required to explore the behavior of the HCCP molecule, which has both organic and inorganic side chains [14]. The P–Cl bond’s strong reactivity makes it possible to introduce a variety of substituents using the appropriate substitution technique [15,16]. Yang et al., 2016 [17] have mentioned the use of cyclotriphosphazene derivatives as a fire retardant. A compound made of phosphorus is varied in its fire-retardant action and thermally quite stable. It is interesting to note that compounds containing phosphorus frequently show both gas and condensed phases [18]. The main goal of this research is to synthesize hexasubstituted cyclotriphosphazene derivatives with Schiff base linkages. The nitrogen atoms in a Schiff base are attached to an aryl or alkyl group by carbon double bonds but not to hydrogen [19]. According to Selvarasu et al., 2016 [20], the stepped core structure of these Schiff base compounds can help to maintain molecular linearity. Additionally, the thermal stability of the Schiff base linkage was subjected to enhance the polymeric matrix’s fire retardant properties [21]. This molecule has the potential to change into a cross-linked structure during burning and in the condensed phase, encouraging the char’s formation on the surface. The char deposits prevent combustion on the polymer matrix’s surface [22]. Additionally, Schiff base molecules have a series of advantages since burning produces gases or vapors with low toxicity and fewer corrosive gases. Other advantages include the low development of smoke in fire incidents and the absence of dioxins [21]. To strengthen the material’s resistance to ignition, HCCP is incorporated into organic material with the linking unit of Schiff base [23]. Consequently, this research assessed the fire-retardant properties of a Schiff base linking unit linked to a cyclotriphosphazene core system.

In this research too, polyurethane was employed as the matrix for the wood coating to examine how well these compounds act as fire retardants. A high-quality cured film produced by polyurethane coating can provide good resistance to solvents and chemicals. Additionally, polyurethane offers desirable qualities including quick setting times, low viscosities and superior flexibility, and their use is expanding quickly in industries such as coatings and paints, clothing and automobiles [24]. These qualities are brought by the primary and secondary structure of polyurethane chains [25]. However, the high flammability of polyurethane materials restricts their use as fire retardants [26]. Due to its low oxygen index, this polyurethane is exceedingly flammable by nature [27]. Hence, the modification of polyurethane coating by incorporating it with cyclotriphosphazene-based compounds with the Schiff base linkage is necessary for overcoming this problem and fully satisfying its specific application.

## 2. Materials and Methods

### 2.1. Chemicals

In this research, the chemicals used were the following: 1-bromopentane, 1-bromotetradecane, 4-hydroxybenzaldehyde, 4-aminophenol, methanol, *N*,*N*-dimethylformamide, potassium iodide, potassium carbonate, anhydrous sodium sulphate, n-hexane, dichloromethane, glacial acetic acid, ethyl acetate, polyurethane sealer, polyurethane lacquer, hardener and thinner. All these solvents and chemicals were used without purification and obtained from Sigma-Aldrich (St. Louis, MO, USA), Merck (Darmstadt, Germany), Acros Organics (Geel, Belgium), BDH laboratory (Poole, UK) and Qrëc (Asia) (Selangor, Malaysia).

### 2.2. Instruments

This research, Thin Layer Chromatography (TLC) was used to identify a product in a mixture and to track the reaction’s progress. The ratios used were 50:50 of ethyl acetate: hexane. FTIR spectroscopy (Bruker, Coventry, UK) was used to characterize the synthesized compounds. The molecular structure of a compound is determined with NMR spectroscopy (Bruker 500 MHz Ultrashield™ spectrometer) for atomic nuclei such as ^1^H, ^13^C and ^31^P. The CHN elemental analysis (CHN analyzer, model PerkinElmer II, 2400, Waltham, MA, USA) is used to calculate the C, H and N percentages in a sample during combustion in an excess of oxygen. Thermogravimetric analysis (TGA), or simply thermogravimetry (TG), is a technique to evaluate the thermal stability of the prepared sample [28]. The thermogravimetric analysis was performed at temperatures ranging from 50 to 700 °C for the HCCP at a heating rate of 10 °C/min under N_2_ flow of 30 mL/min. Moreover, LOI testing (S.S. Instruments Pvt. Ltd., Delhi, India) evaluates the lowest oxygen required to support sample’s combustion. The sample’s preparation was performed by blending with 1 g of compound in 100 mL of polyurethane lacquer after being diluted in methanol. Hardener and thinner were also added to the sample with the ratio of 1:5:5 of polyurethane lacquer, hardener and thinner. Before applying the sample coating onto the wood panel, the sample was stirred homogeneously and the wood panels must be clear of any surface contaminants or imperfections. The final dry film had a thickness of around 90–100 µm, and it was applied using a brush. In order to remove the remnants of solvent, the wood panels were air dried for 10 days and kept in an oven with a temperature of 50–60 °C for two hours before being burned [21]. The LOI testing was executed using an FTT oxygen index, based on BS 2782: Part 1: Method 141 and following standardized tests ISO 4589-1: 2017 and ASTM D: 2863-17a. The wood panel has a dimension of 150 mm × 10 mm × 10 mm. The data will be expressed as percentages (%), and the results of the LOI are calculated using the following given Equation (1):

LOI = *C_F_* + (k × d) (1)

where *C_F_* is the oxygen concentration of the final test, k is factor taken from the manual book *Fire Testing Technology* (ISO 4589) and d is oxygen concentration increment.

### 2.3. Synthesis of the Intermediates and Final Compounds

**Scheme 1:**
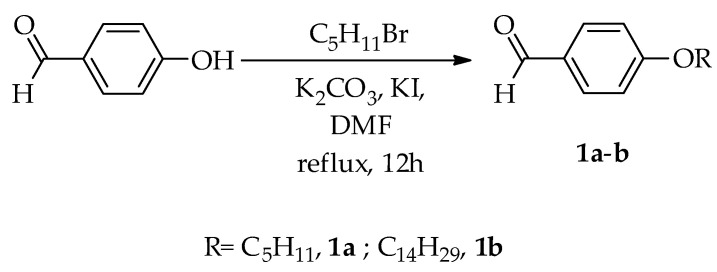
Alkylation reaction of intermediates **1a**-**b**.

**Scheme 2:**
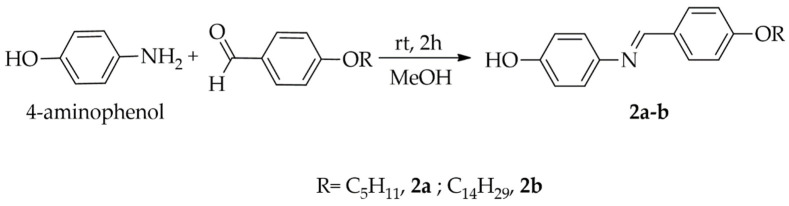
Condensation reaction of intermediates **2a**-**b**.

**Scheme 3:**
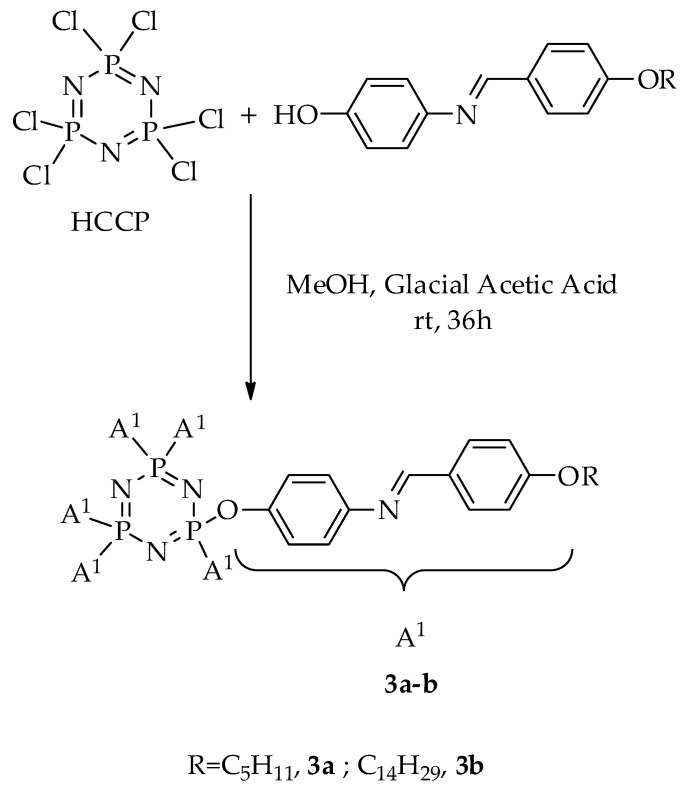
Formation of final compounds **3a**-**b**.

In this research, intermediates **1a** and **1b** was synthesized in Scheme 1 (alkylation reaction) of two types of alkylated bromide (pentyl and tetradecyl) with 4-hydroxybenzaldehyde. Further reaction of 4-aminophenol with intermediates **1a**-**b** in methanol to form intermediates **2a**-**b** (Scheme 2). The reaction of Hexachlorocyclotriphosphazene, N_3_P_3_Cl_6_ with intermediates **2a**-**b** forms final compounds **3a**-**b** as illustrated in Scheme 3.

FTIR spectroscopy, NMR spectroscopy and CHN elemental analysis were used to characterize the synthesized compounds in this study while LOI testing was performed to investigate the compounds’ fire-retardant properties. Section 3.1 contains the whole concise data for the final compounds and intermediates.

## 3. Results

### 3.1. Syntheses

#### 3.1.1. Synthesis of 4-Pentyloxybenzaldehyde, **1a**

In total, 0.1 mol of 4-hydroxybenzaldehyde and 0.1 mol 1-bromononane were separately dissolved in 10 mL of *N*,*N*-dimethylformamide and transferred in a 100 mL round bottom flask. The mixture was added with potassium carbonate (0.15 mol) and potassium iodide (0.01 mol), and it was then refluxed for 12 h. TLC was used to monitor the reaction progress. The mixture was transferred into 250 mL cold water and extracted using ethyl acetate. The organic layers were collected and dried in anhydrous sodium sulphate. The product was filtered, evaporated and dried overnight. The synthesis of **1b** followed the same procedure. Yield: 18.54 g (96.45%), light yellow oil. ^1^NMR (600 MHz, CDCl_3_) δ, ppm: 9.84 (s,1H), 7.79 (d, J = 6.18 Hz, 2H), 6.96 (d, J = 6.84 Hz, 2H), 4.01 (t, J = 4.14 Hz, 2H), 1.77–1.79 (m, 2H), 1.37–1.42 (m, 2H), 0.92 (t, J = 4.80 Hz, 3H). ^13^C-NMR (150 MHz, CDCl_3_) δ, ppm: 190.74, 164.28, 131.97, 129.74, 114.75, 68.40, 28.77, 28.13, 22.44, 14.02.

#### 3.1.2. Synthesis of 4-Tetraloxybenzaldehyde, **1b**

Yield: 26.89 g (84.41%), light yellow oil. ^1^NMR (600 MHz, CDCl_3_) δ, ppm: 9.85 (s,1H), 7.81 (d, J = 8.94 Hz, 2H), 6.97 (d, J = 8.28 Hz, 2H), 4.02 (t, J = 6.18 Hz, 2H), 1.78–1.80 (m, 2H), 1.24–1.44 (m, 2H), 0.87 (t, J = 6.18 Hz, 3H). ^13^C-NMR (150 MHz, CDCl_3_) δ, ppm: 190.85, 164.35, 132.03, 129.77, 114.80, 68.47, 32.00, 29.73, 29.44, 2276, 14.19.

#### 3.1.3. Synthesis of (E)-4-((4-Pentyloxy)benzylidene)amino)phenol, **2a**

A solution of 4-aminophenol (0.02 mol) and intermediate 1a in 40 mL of methanol were mixed in a beaker. The mixture was stirred for about 2 h at room temperature. TLC was used to monitor the reaction’s progress. The mixture was then transferred into cold water (250 mL), filtered and allowed to dry for several days. The synthesis of **2b** followed the same procedures. Yield: 5.05 g (89.18%), light brown powder. ^1^NMR (500 MHz, CDCl_3_) δ, ppm: 8.49 (d, 2H), 7.82 (d, J = 10 Hz, 2H), 7.15 (d, J = 10 Hz, 2H), 7.02 (d, J = 5 Hz, 2H), 6.79 (d, J = 10 Hz, 2H), 4.03 (t, J = 10 Hz, 3H), 1.34–1.75 (m, 2H), 0.88 (t, J = 10 Hz, 3H). ^13^C-NMR (125 MHz, CDCl_3_) δ, ppm: 161.38, 157.06, 156.31, 143.47, 130.42, 129.67, 122.69, 116.12, 115.08, 68.14, 31.69, 25.91, 22.51, 14.40.

#### 3.1.4. Synthesis of (E)-4-((4-Tetraloxy)benzylidene)amino)phenol, **2b**

Yield: 7.16 g (87.45%), light brown powder. ^1^NMR (500 MHz, CDCl_3_) δ, ppm: 8.47 (d, 2H), 7.81 (d, J = 5 Hz, 2H), 7.11 (d, J = 10 Hz, 2H), 7.02 (d, J= 10 Hz, 2H), 6.81 (d, J = 10 Hz, 2H), 4.09 (t, J = 5 Hz, 3H), 1.29–1.79 (m, 2H), 0.90 (t, J = 10 Hz, 3H). ^13^C-NMR (125 MHz, CDCl_3_) δ, ppm: 161.72, 156.82, 156.34, 144.15, 130.29, 129.11, 122.31, 116.39, 115.55, 68.79, 31.61, 29.34, 25.88, 22.28, 13.92.

#### 3.1.5. Synthesis of Hexakis{((E)-4-((4-pentyloxybenzylidene)amino)phenol}triazaphosphazene, **3a**

Hexachlorocyclotriphosphazene, N_3_P_3_Cl_6_ (0.01 mol) was reacted with intermediate **2a** in 40 mL of methanol. Then, as a catalyst, two drops of glacial acetic acid were added to the mixture. The mixture was stirred for about 36 h at room temperature. TLC was used to monitor the reaction’s progress. The mixture was then transferred into cold water (250 mL), filtered and allowed to dry for several days. The synthesis of **3b** followed the same procedures. Yield: 1.70 g (92.83%). Dark green powder. ^1^NMR (500 MHz, CDCl_3_) δ, ppm: 8.44 (d, 2H), 7.93 (d, J = 5 Hz, 2H), 7.50 (d, J = 10 Hz, 2H), 7.14 (d, J = 10 Hz, 2H), 6.80 (d, J = 5 Hz, 2H), 3.92 (t, J = 5 Hz, 2H), 1.25–1.66 (m, 2H), 0.80 (t, J = 5 Hz, 3H). ^13^C-NMR (150 MHz, CDCl_3_) δ, ppm: 161.37, 156.95, 156.32, 143.40, 138.26, 131.55, 129.07, 122.64, 116.14, 68.09, 31.67, 25.89, 22.49, 14.29.

#### 3.1.6. Synthesis of Hexakis{((E)-4-((4-tetraloxybenzylidene)amino)phenol}triazaphosphazene, **3b**

Yield: 2.23 g (86.10%). Light green powder. ^1^NMR (500 MHz, CDCl_3_) δ, ppm: 8.46 (d, 2H), 7.95 (d, J = 10 Hz, 2H), 7.50 (d, J = 5 Hz, 2H), 7.12 (d, J = 10 Hz, 2H), 6.81 (d, J = 5 Hz, 2H), 4.04 (t, J = 5 Hz, 2H), 1.25–1.76 (m, 2H), 0.87 (t, J = 10 Hz, 3H). ^13^C-NMR (150 MHz, CDCl_3_) δ, ppm: 161.37, 156.95, 156.32, 143.40, 138.26, 131.55, 129.07, 122.64, 116.14, 68.09, 31.67, 25.89, 22.49, 14.29.

The overall FTIR results of compounds **1a**-**b** (Scheme 1), **2a**-**b** (Scheme 2) and **3a**-**b** (Scheme 3) were summarized in Table 1.

### 3.2. CHN Elemental Analysis

The data for the compound’s elemental analyses (**1b**, **2a**-**b** and **3a**-**b)** were summarized in Table 2. The purity of the tested samples has a significant impact on the precision and accuracy of results in the CHN analyzer. Poor sample preparation can result in solvent contaminants, sampling errors and moisture residues that are left after any action with the tested sample, while volatility can also lead to changes in composition and heterogeneity. The results of the element analysis solvent could be invalidated by all of the aforementioned [29].

The successful execution of the synthesis method and the products’ purification was confirmed by the decent agreement between theoretical values and experimental values of carbon, hydrogen and nitrogen.

## 4. Discussion

### 4.1. FTIR Spectral Data of the Intermediates and Final Compounds

This research involved the alkylation reaction between alkylbromide and 4-hydroxybenzaldehyde in the presence of potassium iodide and potassium carbonate to form intermediates **1a** and **1b** (Scheme 1). The intermediates and compounds analyses were performed on the Bruker Spectrum, 4000–400 cm^−1^ FT-IR/FT-NIR Spectroscopy. Even though intermediates **1a** and **1b** differed in the number of alkyl chains, they revealed a similar pattern. The IR data for intermediates **1a** showed two bands at 2955 and 2860 cm^−1^ (Csp^3^–H stretching). The bands at 1687 and 1599 cm^−1^ were assigned to the C=O and C=C stretching, while the absorption band at 2734 cm^−1^ was attributed to the aldehydic C–H stretching. The absence of O–H stretching at 3300 cm^−1^ confirmed the successful insertion of the alkyl group into benzaldehyde.

**Figure 1:**
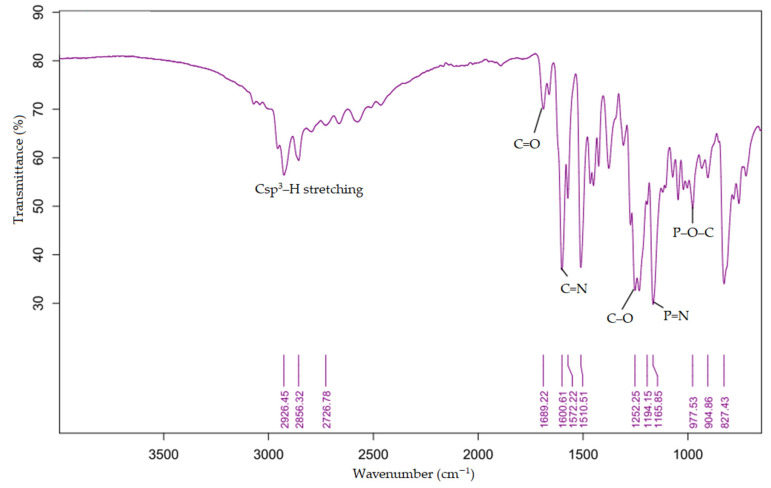
Fourier transform Infra-Red (FTIR) spectrum of compound **3a**.

Further reaction of intermediates **1a** (pentyl chain) and **1b** (tetradecyl chain) with 4-aminophenol produced two Schiff base compounds, **2a** and **2b,** with different substituents (Scheme 2). The condensation reaction of 4-aminophenol with intermediates **1a** and **1b** formed intermediates **2a** and **2b** with Schiff base linking units. The azomethine group (C=N) of intermediates **2a** could be seen at 1604 cm^−1^. The band at 1688 cm^−1^ showed the absence of carbonyl group (C=O), indicating the reaction was a success. The bands at 2926 and 2854 cm^−1^ were attributed to Csp^3^–H stretching. The bands at 1571 cm^−1^ were allocated to C=C stretching, while the band at 1253 cm^−1^ was responsible for the C–O stretching. Intermediate **2a**-**b** reacted with HCCP to form final compounds **3a**-**b** with a Schiff base linking unit (Scheme 3). Based on the IR spectra of compound **3a** (Figure 1), 2926 and 2856 cm^−1^ absorption bands were the Csp^3^–H stretching and correspond to the alkoxy moieties in the compound. The aromatic C=C and C–O stretching were observed in the absorption bands of 1510 and 1252 cm^−1^, respectively. The appearance of P–O–C bending was at 977 cm^−1^, while the absorption bands of P=N stretching were observed at 1165 cm^−1^. The azomethine linkage (C=N) was at 1600 cm^−1^ and the disappearance of the hydroxy (–OH) group in the IR spectra showed that the hexasubstituted cyclotriphosphazene in the side arms was successfully formed.

### 4.2. NMR Spectral Discussion Final Compounds

**Figure 2:**
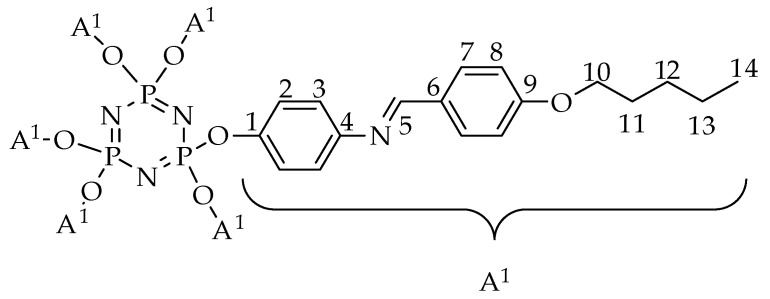
Structure of compound **3a** with complete atomic numbering.

The confirmation of structure in the series was represented by compound **3a,** and the complete atomic numbering of compound **3a** was shown in Figure 2.

**Figure 3:**
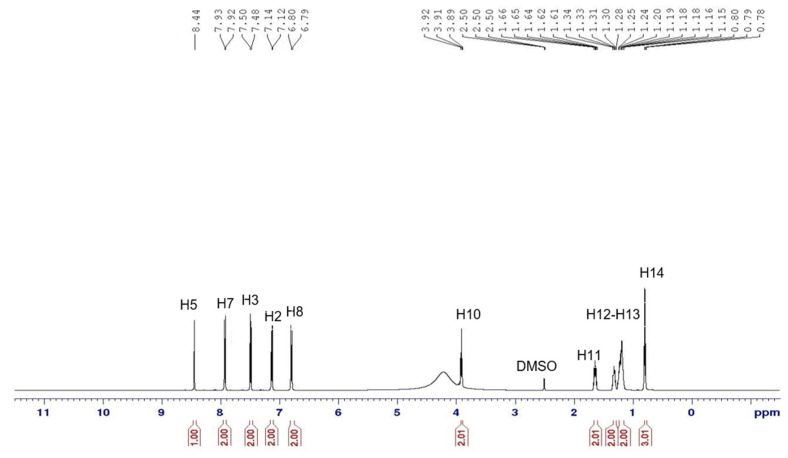
^1^H-Nuclear magnetic resonance (NMR) spectrum of compound **3a**.

Based on Figure 3, the final compound **3a** ^1^H-NMR spectrum showed azomethine proton signals (Schiff base linkage) seen at δ 8.44 ppm (**H5**) in the downfield region. The presence of the Schiff base group leads the (**H7**) signal to be the utmost deshielded aromatic protons at δ 7.93 ppm. The other distinguishable doublets of different aromatic protons were detected at δ 6.80, 7.14 and 7.50 ppm and assigned as **H8**, **H2** and **H3**, respectively. Among other methylene protons, the oxymethylene protons (**H10**) were deshielded due to their close proximity to the electronegative oxygen atom. Hence, the signal of these protons was seen at δ 3.92 ppm as a triplet, while that of the other methylene protons **H11** appeared as a multiplet at δ 1.66 ppm and **H12**-**H13** also showed the multiplet in δ 1.34-1.25 ppm region. In the most upfield region, a triplet of **H14** appeared at δ 0.80 ppm.

**Figure 4:**
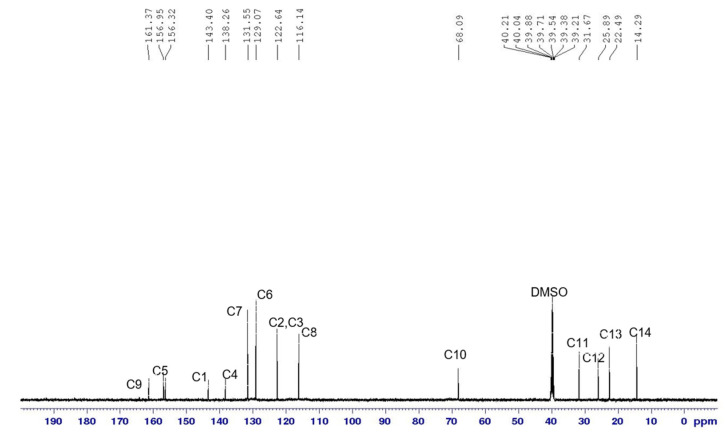
^13^C NMR spectrum of compound **3a**.

The ^13^C NMR spectrum of final compound **3a,** which can be observed in Figure 4, designates that **3a** has a total of 14 carbon signals in the side arms. These signals contain one azomethine, two quaternary, two aromatics, one methyl carbons and six methylene.

**Figure 5:**
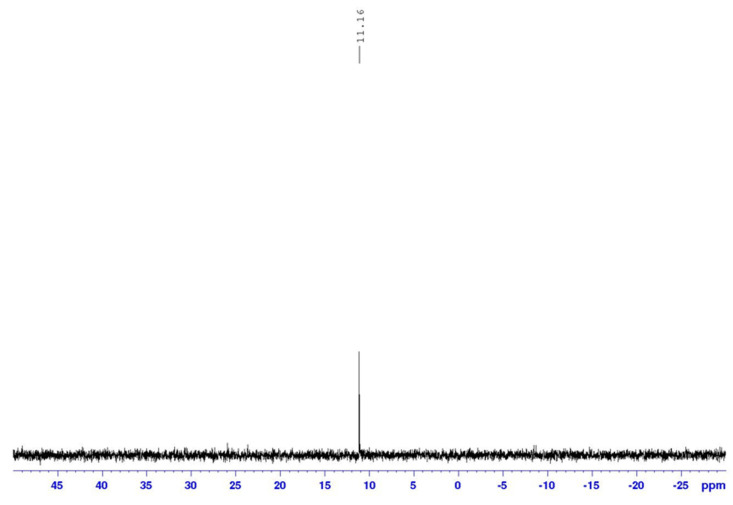
^31^P NMR spectrum of final compound **3a**.

The phosphorus signal at 11.16 ppm, which appeared as a singlet in the ^31^P NMR spectra of compound **3a** (Figure 5), corresponds to all the phosphorus in the cyclotriphosphazene ring containing similar substituents in the side arms.

### 4.3. Determination of Thermal Property

**Figure 6:**
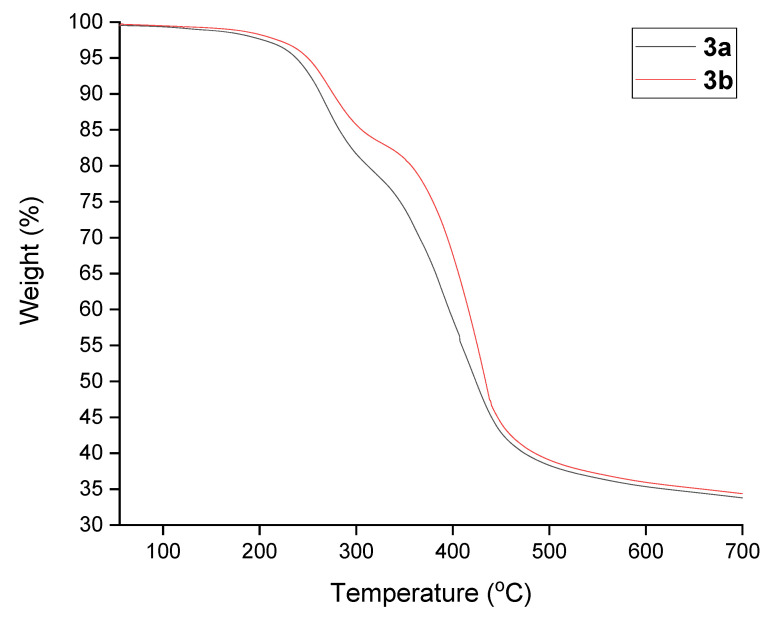
TGA curves of compounds **3a** and **3b** from 50 to 700 °C at a heating rate of 10 °C/min under N_2_ flow of 30 mL/min.

Cyclotriphosphazene, consisting of alternating phosphorus and nitrogen atoms, exhibits unusual thermal stability [30]. TGA was utilized to determine the thermal stability of the cyclotriphosphazene ring linked to the Schiff base group. The thermal stability of HCCP compounds was shown in Figure 6. A stack plot of TGA curves of **3a** and **3b** with a flow of 30 mL/min nitrogen shows good thermal stability. Both curves display two distinctive weight loss stages. Compound **3a** begins to decompose first at 260 °C, and the second phase of weight loss was at 370 °C and it was totally degraded at 460 °C while compound **3b** was stable up to 280 °C in the first phase and the second phase of weight loss was at 400 °C. Compound **3b** totally degraded at 480 °C. This result may be beneficial for fire-retarding compounds that undergo combustion at relatively high temperatures [21]. The initial degradation temperature of compound **3a** is slightly lower than that of compound **3b,** and this might be attributed to the longer alkyl chain of compound **3b**. Besides, the second phase of decomposition of both compounds was observed due to the formation of char layers in the molecules [31]. Based on TGA curves in Figure 6, the char layer yield for compounds **3a** and **3b** is 33.77 and 34.33%, respectively. This phenomenon indicates that both compounds can form the charring process at high temperatures, which is attributed to the presence of a cyclotriphosphazene ring system, linking units and terminal groups at the side arm [32].

Both HCCP compounds show excellent thermal stability due to the occurrence of chemically stable P–N skeletons and cross-linking structures [11]. The lower degradation temperature of HCCP compounds can be attributed to the fact that the P–O–C linkage is easier to decompose. This helps in forming a char layer for the protection of the wood matrix [33]. In addition to that, this could also be attributed to the ability of Schiff’s bases based on p-hydroxybenzaldehyde to form an intrahydrogen bond with the azomethine group, which is reflected in the thermal stability of HCCP compounds [21].

### 4.4. Determination of Fire Retardant Property

Hexasubstituted cyclotriphosphazene compounds were physically added as additives into the polyurethane coating. The LOI test was used to verify the fire-retardant properties of the HCCP compounds, and polyurethane was employed as a matrix for wood coating. Polyurethane was chosen due to its preferable characteristics such as good flexibility, poor viscosity, high setting speeds and good thermal stability [24]. However, due to the high flammability of polyurethane materials restricts their use in daily life [26]. By using only 1 wt.%, the preparation of the sample was performed to reach the highest fire retardancy with a minimum amount of additive. The purpose is to minimize the starting of a fire by providing ignition resistance and slowing the spread of fire. The LOI method is frequently used to assess the fire retardancy of samples. Compounds with LOI values of 25% or more are regarded as self-extinguishing [34]. For LOI testing, the samples were vertically suspended inside a closed chamber and equipped with inlets of nitrogen and oxygen gas in order to achieve a controlled environment. Starting from the top, the sample was burned and the environment was changed to control the required oxygen to burn the sample at specific periods [35].

Based on the summarized LOI results in Table 3, polyurethane has a low oxygen index value, which is comparable to the LOI value of conventional polyurethane reported previously [27,36]. The LOI value of polyurethane coating at 21.90% was increased to 24.42% when 1 wt.% of compound **3a** was incorporated into the coating and increased more when incorporated with compound **3b** (24.90%). The results indicated that even only a minimum number of additives added could improve the LOI value of polyurethane. The LOI results are also positively influenced by HCCP, which has six chlorine atoms that induce good fire retardancy and thermal stability [37]. This performance was due to its high phosphorus content and the hexa-functionality of the HCCP compound [38]. The LOI value responded positively to the HCCP’s modification with an organic intermediate. In addition to that, it was discovered that Schiff base linking units improved the fire-retardant properties. In the condensed phase, the cross-linked structure of Schiff base compounds provides great thermal stability and encourages the production of char on the surface [21,39]. Wang et al., 2020 [26] also reported the hexa-[4-[(2-hydroxy-ethylimino)-methyl]-phenoxyl]-cyclotriphosphazene (HEPCP) that gives a higher LOI value due to the synergistic effect of cyclotriphosphazene and Schiff base group. According to Table 3, the increase in values of LOI was due to the increase in alkyl chain length. Compound **3b,** with the longest alkyl chain, gives the highest LOI value. This happened because high-molecular-weight compounds offered greater fire retardant properties compared to low-molecular-weight compounds [21,40]. According to earlier research, the synergistic interaction between the fire-retardant properties of phosphorus and nitrogen increases the thermal properties and fire retardancy of cyclotriphosphazene with an alkyl chain [41,42].

## 5. Conclusions

A series of hexasubstituted cyclotriphosphazene compounds with pentyl and tetradecyl substituents were successfully synthesized. The characterization of these compounds was performed using spectroscopic techniques of Fourier Transform infrared (FT-IR), ^1^H, ^13^C and ^31^P nuclear magnetic resonance (NMR) and CHN elemental analysis. The signal for Schiff base linkage appeared at 1600 cm^−1^ and δ 8.44 ppm in the FTIR and ^1^H-NMR spectra, respectively. The presence of a singlet at 11.00 ppm in the ^31^P-NMR showed the success of the hexa-functionality of side arms. Both HCCP compounds showed good thermal stability due to the P-N linkage and the structure cross-linked of cyclotriphosphazene, but compound **3b** showed slightly better thermal stability due to the longer C-C bond. The prepared HCCP compound was then added physically into polyurethane formulations for surface coating application of wood, and thus further research on the fire retardancy of **3a**-**3b** was conducted using the LOI test. The addition of compound **3b** improved the fire-retardant property of the polyurethane coating, thus giving the highest LOI value in this experiment of 24.90%. This was due to the synergistic and high molecular weight effects towards greater fire-retardant properties.

## Figures and Tables

**Table 1:** FTIR results of compounds **1a**-**1b** (Scheme 1), **2a**-**2b** (Scheme 2) and **3a**-**3b** (Scheme 3).

Vibrational (Stretching, cm^−1^)										
Scheme	Compound	O–H	C–H	Csp^3^–H	C=O	C=C	C–O	C=N	P=N	P–O–C
**1**	**1a**	-	2734	2955, 2860	1687	1599	1253	-	-	-
	**1b**	-	2733	2914, 2847	1689	1599	1250	-	-	-
**2**	**2a**	3069	-	2926, 2854	-	1571	1253	1604	-	-
	**2b**	3437	-	2915, 2848	-	1577	1250	1606	-	-
**3**	**3a**	-	-	2926, 2856	1689	1572	1252	1600	1165	977
	**3b**	-	-	2915, 2848	1691	1572	1249	1600	1169	972

**Table 2:** CHN elemental analysis data of compounds **1b**, **2a**-**b** and **3a**-**b**.

Compound	% Found (Calculated)		
	% C	% H	% N
C_21_H_34_O_2_, **1b**	78.84 (79.19)	10.67 (10.76)	-
C_18_H_21_NO_2_, **2a**	76.18 (76.29)	7.40 (7.47)	4.81 (4.94)
C_27_H_39_NO_2_, **2b**	78.99 (79.17)	9.51 (9.60)	3.36 (3.42)
C_108_H_120_N_9_O_12_P_3_, **3a**	70.78 (70.92)	6.55 (6.61)	6.80 (6.89)
C_162_H_228_N_9_O_12_P_3_, **3b**	75.16 (75.23)	8.80 (8.88)	4.81 (4.87)

**Table 3:** LOI test results.

Compound	LOI Value (%)
Blank Polyurethane	21.90 (± 0.00)
Compound **3a**	24.42 (± 0.00)
Compound **3b**	24.90 (± 0.00)

## Data Availability

The data presented in this study are available on request from the corresponding author.
